# Use of Solar Panels for Shade for Holstein Heifers

**DOI:** 10.3390/ani13030329

**Published:** 2023-01-17

**Authors:** Ana Flávia P. A. Faria, Alex S. C. Maia, Gustavo A. B. Moura, Vinícius F. C. Fonsêca, Sheila T. Nascimento, Hugo F. M. Milan, Kifle G. Gebremedhin

**Affiliations:** 1 Innovation in Thermal Comfort and Animal Welfare (Inobio-Manera), Animal Biometeorology Laboratory, São Paulo State University, Jaboticabal 14884-900, SP, Brazil; 2 Brain Function Research Group, School of Physiology, University of the Witwatersrand, Johannesburg 2193, Gauteng, South Africa; 3 Innovation in Thermal Comfort and Animal Welfare (Inobio-Manera), Maringá State University, Maringá 87020-900, PR, Brazil; 4 Department of Biological and Environmental Engineering, Cornell University, Ithaca, NY 14853, USA

**Keywords:** animal agrivoltaics, dairy cattle, shade, thermal comfort

## Abstract

**Simple Summary:**

Using photovoltaic panels to provide artificial shade for animals can result in the “co-generation” of electrical energy, and it is an efficient way of achieving net-zero methane emission and reducing one of the most powerful greenhouse gases (GHG). This concept and practice is termed “Animal Agrivoltaics”. In this study, we examined the impacts of Animal Agrivoltaics on the thermal comfort and wellbeing experienced by dairy heifers, and the potential benefit of offsetting enteric methane emissions (*e*CH_4_, g animal^−1^ day^−1^). The shade provided by the solar panels efficiently relieved the heat load on the heifers, cooled off their body surface and skin temperatures, and decreased the costs of thermoregulation, as indicated by the lower requirement of panting. Based on the mean daily methane emission rate and the amount of equivalent CO_2_ (CO_2-eq,_ g day^−1^) that was not emitted into the atmosphere due to electricity generated by the solar panels, 4.1 m^2^ of solar panels would be necessary to offset the *e*CH_4_ emitted by the heifers.

**Abstract:**

Animal Agrivoltaics combines electric energy generation, animal thermal comfort, and sustainable production at the same time. This model of production can foster the sustainable intensification of dairy production in tropical areas where solar irradiance is high and nearly constant throughout the year. In this study, we propose Animal Agrivoltaics as an alternative practice to reduce the heat load and *e*CH_4_ emissions from dairy heifers in tropical areas. To attest this hypothesis, (1) the meteorological data and the behavioral and physiological responses of the animals were integrated in order to determine the benefits provided by the shade from the solar panels on the thermoregulation of the dairy heifers, and (2) measurements of the enteric methane emissions were taken to determine the potential of the solar panels to offset the GHG. Seven crossbred Holstein heifers (7/8, Holstein × Gyr) with a mean body weight of 242 kg (SD = 53.5) were evaluated in a paddock shaded with ten modules of solar panels. Miniature temperature loggers were used to record the body surface, skin and vaginal temperatures of the heifers every five minutes. The respiratory rate and the shade-use behavior were also monitored by two observers. These measurements were taken from 08:00 to 17:00 h for 18 consecutive days. After completing the field study, the heifers underwent for assessments of the daily oscillations of *e*CH_4_ emission using a flow-through respirometry system. The use of shade by the heifers was progressively increased (*p* < 0.01) with an increasing level of solar irradiance. Lying and ruminating were more likely (*p* < 0.01) to occur when the heifers were in the shade, especially when the solar irradiance exceeded 500 W m^−2^. Between 10:00 and 14:00 h, the heifers benefited from the shade produced by the solar panels, with a reduction of 40% in the radiant heat load. With an increasing intensity of solar irradiance, body surface temperature, skin temperature and respiratory rate of the heifers in the shade were lower (*p* < 0.01) compared to when they were exposed to the sun. The heifers had a daily methane emission total of 63.5 g per animal^−1^ or 1.7 kg of CO_2-eq_. Based on this emission rate and the amount of CO_2-eq_ that was not emitted to the atmosphere due to the electricity generated by solar panels, 4.1 m^2^ of panels per animal (nominal power = 335 W) would be expected to obtain a net-zero *e*CH_4_ emission. Over a period of one year (from September 2018 to August 2019), a set of ten photovoltaic panels used in the study produced 4869.4 kWh of electricity, thereby saving US $970.00 or US $48.00 per m^2^ of solar panel. Based on the results of this study, it can be concluded that use of Animal Agrivoltaics, in addition to producing electricity, has significant potential benefit in providing better thermal comfort to cattle, as well as offsetting the enteric methane emissions released into the environment. In addition, the system would provide extra income to farmers, as well as a potential source of energy micro-generation.

## 1. Introduction

The rapid change in the thermal environment has been detrimental to livestock’s comfort, production and economic outcomes [1]. On one hand, there is the expected increase in world population (it is expected to be 9.7 billion in 2050), which will require a dramatic increase in food security, while on the other hand, societal concerns about animal welfare and the environmental responsibility issues of farming practices are mounting. In some part of the world, livestock farming systems are being forced to reduce their greenhouse gas emissions (GHG), especially methane [2]. Thus, to ameliorate the negative impacts of increasing heat load on animals due to climate change and to increase the demand for food in a sustainable way, it will be necessary to: (1) improve the efficiency of feed conversion into animal production and (2) employ economically sustainable adaptation practices to improve the thermal comfort and wellbeing of the animals. 

The majority of the 12 million Brazilian dairy cows that produce 34 billion liters of milk per year are managed in open-pasture fields or paddocks located between a southern latitude of 5° and 20° [3]. In this range of latitude, the solar irradiance is above 800 W m^−2^, and it has been reported that the heat load on cows could be as much as 650 W m^−2^ [4]. This amount of heat load is as much as threefold that of metabolic heat production of a cow (∼200 W m^−2^) [5]. To maintain thermal equilibrium, a cow would need to evaporate up to 260 g h m^−2^ or 1300 g h^−1^ animal^−1^ of sweat. In such circumstances, the thermal comfort, health, reproduction and production performance of cows would be impaired if they do not have access to shade [6,7]. 

Providing shade for dairy cows is a necessity in a tropical climate. From a positive standpoint, the high level of solar irradiance can be used to generate renewable electrical energy through photoelectric cells, a concept that is named Animal Agrivoltaics. Animal Agrivoltaics can be considered to be a co-generation system where energy and food can be produced in the same area. The first published findings on this topic demonstrated that Animal Agrivoltaics is a viable option for raising sheep in tropical areas [8] because the shade from solar panels efficiently reduced the heat load on the animals, and increased the frequency of shade seeking, it increased the frequencies of lying down and rumination. However, its effectiveness in providing thermal benefits to animals, i.e., cooling down their body temperatures and reducing the costs of thermoregulation still need to be investigated. Over a one year period, other benefits were had, for example, 5.19 MWh of electricity was produced, which is a monetary saving of US $740, and additionally, 2.77 tons of CO_2_ was not emitted to the atmosphere [8]. In terms of the reduction of other GHGs from livestock systems, Animal Agrivoltaics has the potential to offset the enteric methane emissions from cattle [9]. 

This study proposes Animal Agrivoltaics as a viable alternative for the sustainable management of dairy production systems in tropical areas. The system ameliorates the solar input on heifers, offsets the enteric methane emissions, generates renewable energy, has the potential to be micro-generator of electricity, and it could be a source of income to farmers. To test this hypothesis, the meteorological data and the behavioral and physiological responses of the animals were continuously measured and integrated in order to determine the benefits of Animal Agrivoltaics on the thermoregulation of heifers. In addition, measurements of enteric methane were taken to determine the potential of Animal Agrivoltaics for mitigating the GHG.

## 2. Materials and Methods

### 2.1. Animals and Experimental Design

**Figure 1:**
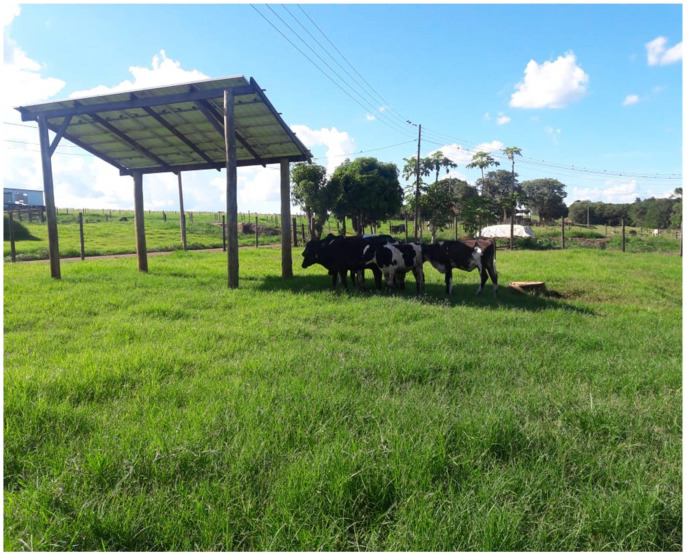
Crossbred Holstein heifers in the experimental paddock seeking shade produced by solar panels.

The procedures involving the animals were approved by the Animal Ethics Committee of the São Paulo State University, Jaboticabal, Sao Paulo, Brazil (013070/2018). Seven healthy crossbred Holstein heifers (7/8, Holstein × Gyr) with mean body mass of 242 kg (SD = 53.5) and an age of 10 months were used in this study. Approximately four weeks before the beginning of the study, the heifers were treated with anthelmintic medication (1 mL 20 kg BW of 10% fenbendazole, MSD Saúde Animal, São Paulo, Brazil), and immunized against clostridia (5 mL animal^−1^; Valée S/A Produtos Veterinários, Montes Claros, Brazil). The behaviors and physiological responses of the heifers were monitored for 18 consecutive days (from 2 to 20 April 2019). The heifers were kept in a paddock (Area = 1.900 m^2^) with Cynodon grass in the Animal Biometeorology Laboratory at the Sao Paulo State University, Jaboticabal, Sao Paulo, Brazil. The feed supplement was a pre-formulated diet consisting of 30% roughage (corn silage) and 70% of concentrate (76% corn, 7% soybean, 8% wheat bran, 5% cottonseed meal, 3% mineral core and 1% of dicalcium phosphate), and it was given daily at 17:30 h. Water was freely available. The paddock was shaded with a roof structure that consisted of ten photovoltaic panels (1.0 m × 2.0 m width × length; 335 Wp, peak efficiency of 16.72%, Canadian Solar model CS6Ue335P, Guelph, ON; installed by Blue Sol, Blue Sol Anergia Solar, Ribeirão Preto, SP), with the following dimensions: a lower height of 3.0 m; an inclination angle of 15°; a width of 4.0 m; a length of 5.0 m; an area of 20 m^2^; a total shade area of 19.3 m^2^ or 2.76 m^2^ animal^−1^ (Figure 1). 

### 2.2. Meteorological Variables

The meteorological variables including the air temperature (T_A_, °C; accuracy ±0.5 °C; range: from −35 to 50 °C), relative humidity (R_H_, %; accuracy ±3%; range: from 0 to 100%), black globe temperature in the sun (T_G_, °C; accuracy ±0.1 °C), wind velocity (U, m s^−1^; accuracy ±0.44) and direction (U_d_, degrees; accuracy of ±3%; range: from 0° to 360° degrees), solar irradiance (R_S_, W m^−2^; CMP-22, Kipp and Zonen, Delft, The Netherlands; spectral range: 0.3–3.6 µm) and ultraviolet solar irradiance (R_UV_, W m^−2^; CMP-22, Kipp and Zonen, Delft, The Netherlands; spectral range: 0.28–0.4 µm) were recorded every 1 min using a portable weather station (Campbell Scientific CR10X Model, Logan, UT, USA) placed near the (~1 m) paddock.

### 2.3. Thermal Stress Indicators

#### 2.3.1. Body Temperatures

The body temperatures including the vaginal (T_V_, °C; Star-Oddi, Reykjavik, Iceland, DST centi-T, accuracy ±0.1 °C, range: from 5 to 45 °C), skin (T_skin_, °C; iButton DS1921; Maxim Integrate, San Jose, CA, USA, accuracy ±0.5 °C, and range: from −40 to 85 °C) and body surface temperature (T_S_, °C; iButton DS1921; Maxim Integrate, San Jose, CA, USA, accuracy ±0.5 °C and range from −40 to 85 °C) were remotely measured every five minutes using temperature loggers attached to the heifers. For the vaginal temperature, T_V_, a temperature logger was intravaginally attached using a modified vaginal, hormone-free controlled internal drug release insert (CIDRTM; InterAg, Hamilton, New Zealand). For T_skin_ and T_S,_ two other loggers were attached between the second and third most posterior ribs, always in a black-spotted body region (20 × 20 cm) using surgical tape (3M Micropore, St. Paul, MN, USA). A 10 × 10 cm region was shaved in order to attach the T_skin_ logger. All of the loggers were calibrated at 2 °C increments between 30 and 42 °C in a thermally insulated box against a highly accurate thermocouple (Type K; temperature range = from −40 to 1300 °C; accuracy ±0.2 °C). During the experimental period, the loggers were removed, and the data were retrieved every seven days. The respiratory rates (R_R_, breath min^−1^) were measured every 30 min daily by two observers from 08:00 to 17:00 h. The agreement between the observers was above 0.95, as measured by Pearson correlation. 

#### 2.3.2. Shade-Use Behavior

Two observers scanned the behaviors of the heifers every five minutes [10] to observe the shade-use behaviors. The lying or standing of the heifers were recorded whether the heifers were ruminating or not. The inter-observer agreement was above 0.99, as measured by the Pearson correlation. An animal was in shade when, at least, her head or one of her hooves was in the shade, otherwise, it would be in the sun [8]. Ruminating was defined as chewing movements without there being feed in the mouth, feed regurgitation or both of them [11], otherwise, idling was recorded. Lying was defined as when the flank of a heifer was in direct contact with the ground, otherwise, it was considered to be standing. 

#### 2.3.3. Thermal Evaluation of Shade of Solar Panels

Thermal evaluations of shade of the solar panels were performed using the Radiant Heat Load (R_HL_, W m^−2^) [12] as follows:

R_HL_ = σ (T_MR_)^4^

where σ is the Stefan–Boltzmann constant 5.67 × 10^−8^ (W m^−2^ K^−4^); T_MR_ (K) is the mean radiant temperature calculated from black globe temperature. A black globe device was placed one meter above the ground surface in the shade of the solar panels. A temperature data logger (i-bottom DS1925L, Maxim Integrated, Sao Jose, CA, USA; size = 0.60 × 1.70 cm, height × diameter; accuracy ±0.5 °C) was inserted inside the globe for measuring the black globe temperature in the shade (T_Gshade_, °C). The black globe temperature for the sun (T_Gsun_, °C) was obtained from the weather station. Five temperature data loggers were attached to the inner surface of the solar panels to obtain the mean inner surface temperature of the solar panel (T_SP,_ °C; i-bottom DS1925L, Maxim Integrated, Sao Jose, CA, USA size = 0.60 × 1.70 cm, height × diameter; accuracy ±0.5 °C). 

#### 2.3.4. Generation of Electricity, CO_2_ and CH_4_ Savings

Between September 2018 and August 2019, the electricity generated by the photovoltaic panels was recorded every five minutes using a frequency inverter (Fronius 3 kWp). The amount of CO_2_ that was not emitted into the atmosphere was calculated using the 2018 and 2019 daily Brazilian CO_2_ emission factor for electric energy generation (for hydroelectric, wind, photovoltaic and thermal power) [13,14,15]. After completing the field study, six heifers were assigned to a Latin square design for an assessment of their enteric methane emissions over twelve consecutive days. A flow-through respirometry system with a non-ventilated face mask was used [16,17]. The hourly oscillations of CH_4_ (VCH_4_, L h^−1^) were obtained to determine the daily CH_4_ emission rates (*e*CH_4_, g animal^−1^ day^−1^). The *e*CH_4_ was then multiplied by a factor of 27 to obtain the equivalent CO_2_ rates (CO_2-eq_, g animal^−1^ day^−1^). The amount of *e*CH_4_ mitigated by Animal Agrivoltaics was determined by comparing the *e*CH_4_ emitted by heifers (transformed to CO_2-eq_) to the amount of CO_2_ that was not emitted into the atmosphere due to electric generation by the solar panels. 

### 2.4. Statistical Analyses

Confirmatory models were fitted by applying conventional statistical techniques through a mixed model based on Generalized Least Squares (GLS) using the Statistical Analysis System (SAS Institute, Version 8). Because of the repeated nature of the data (e.g., body temperatures and respiratory rate), the covariance structure of the model was chosen carefully [18]. Different covariance structures were tested (compound symmetry, first-order auto regression, Toeplitz, first-order ante-dependence ones and others), and the best covariance structure was chosen based on the Akaike’s information criterion (AIC). The best-fitted models to predict the body temperatures and respiratory rate were based on the following independent variables:

Y_ijklmn_ = µ + L*_i_* + D*_j_* + A_k_ + R*_l_* + P*_m_* + R*_l_* (L*P)*_im_* + ε_ijklmn_

where Y_ijklmn_ is the dependent variable; L is the fixed effect in the *i*th location (j = Shade or exposed to sun); D is the fixed effect of *j*th day of the evaluation; A is the fixed effect of the *k*th animal (k = 1, 2, 3,…,7); R is the fixed effect of the *l*th class of solar irradiance (if 100 ≤ R_S_, then m = class 200; if 200 < R_S_ ≥ 300, then m = class 300,…; if 900 < R_S_, then m = 900); *p* is the fixed effect of the *m*th period of the day (if 07:00 h < time of the day < 12:00 h, then m = morning; if 12:00 h < time of the day ≥ 17:00 h, then m = afternoon). The μ is the parametric mean and ε_ijklmn_ is the residual term. The shade-use behavior was analyzed using nonparametric regression analyses through the Generalized Additive Models (GAM Procedure; Binary distribution) by using the air temperature, black globe temperature, solar irradiance and wind speed as independent variables. Generalized mixed models (Mixed Procedure) were used to predict the daily enteric methane emissions, which included the time of day and the number of days of evaluation as fixed factors, and the animals were the random factors.

## 3. Results and Discussion

**Figure 2:**
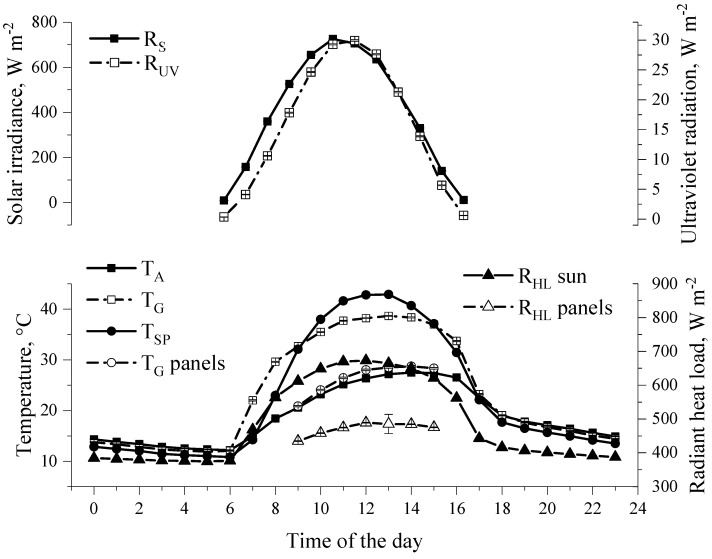
Least square means (±SEM) of the meteorological variables during thermal evaluations of the shade of solar panels. T_SP_ = surface temperature of panels; T_Gpanels_ = black globe temperature in shade projected by solar panels; TG = black globe temperature in full sun; R_HL_panels = Radiant heat load in shade of panels; R_HL_sun = Radiant heat load in full sun.

**Figure 3:**
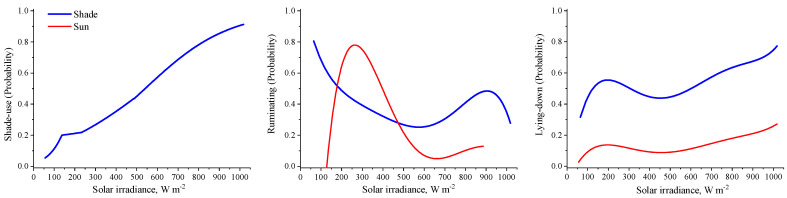
Probability of shade use of heifers as a function of solar irradiance.

This study confirmed the initial hypothesis that the shade produced by the solar panels is an efficient alternative in reducing the heat load and costs of thermoregulation (Figure 2) for animals, in addition to offsetting the enteric methane emissions and generating extra income for farmers. Overall, our results revealed that the heifers were more likely to be in shade when the solar irradiance exceeded 700 W m^−2^ (Figure 3). This magnitude of solar irradiance was also reported to be the threshold for the shade-seeking behavior of Holstein cows managed in tropical pastures [19,20]. In addition to protecting the heifers from direct short-wave solar radiation, the heifers relieved 40% of their heat load (R_HL_, W m^−2^) by being in the shade (Figure 2). When comparing different types of shading structures, our previous investigation on Animal Agrivoltaics with sheep showed that the shade of the solar panels lowered the R_HL_ by 30% compared to that of the conventional shade-cloth structure [8]. The R_HL_ accounts for the radiant heat exchange between the heifers and the surrounding environment [4]. 

The positive outcome of shade-use behavior for animals is also the abatement of direct short-wave solar radiation. By seeking shade, the heifers were able to avoid short-wave solar load in the amount of 1000 W m^−2^ (Table 1). On a clear sky day, the direct solar component represents 80% of the total short-wave input [3]. The shade also serves as a heat sink for animals, especially when it is not projected below the roof structure. The efficiency of the energy conversion of photovoltaic cells ranges between 15 and 25%. Part of the solar beam that is intercepted by the solar panels is absorbed as heat, thus increasing the temperature of the external and inner surfaces of the solar modules. Between 10:00 and 14:00 h, the temperature of the inner surface of the solar panels was between 35 and 38 °C. During this time interval, the solar elevation angle is near the zenith, and the shade is projected below the solar panels. Under such circumstances, if the body surface temperature of the heifers is below 35 °C, then the balance of the thermal radiation between the animals and the solar panels results in a net gain of long-wave radiation in favor of the animals. On the other hand, when the shade is projected and exposed to a clear sky (Figure 1), the animals that are in shade experience more thermal comfort because the temperature of the clear sky would be lower than the temperature of the terrestrial surfaces [4,21].

The heifers in this study also lost heat by conduction by lying down on a shaded, cool surface. They also were exposed to less long-wave radiation emitted from the ground surface. Because lying and ruminating were more likely to occur (*p* < 0.05) when the animals were in the shade (Figure 3), especially if solar irradiance is above 500 W m^−2^, it is reasonable to assume that the heifers were thermally comfortable. When animals face heat stress, the frequency of lying and ruminating behavior of cows are reduced [22]. When they are grazing in a tropical climate, at times of high radiant heat load, cows avoid lying down on hot, sunny surfaces to avoid heat gain by conduction [4]. Previous studies with sheep also confirmed that the shade provided by solar panels increased the frequency of shade seeking, lying and rumination behaviors [8]. 

**Figure 4:**
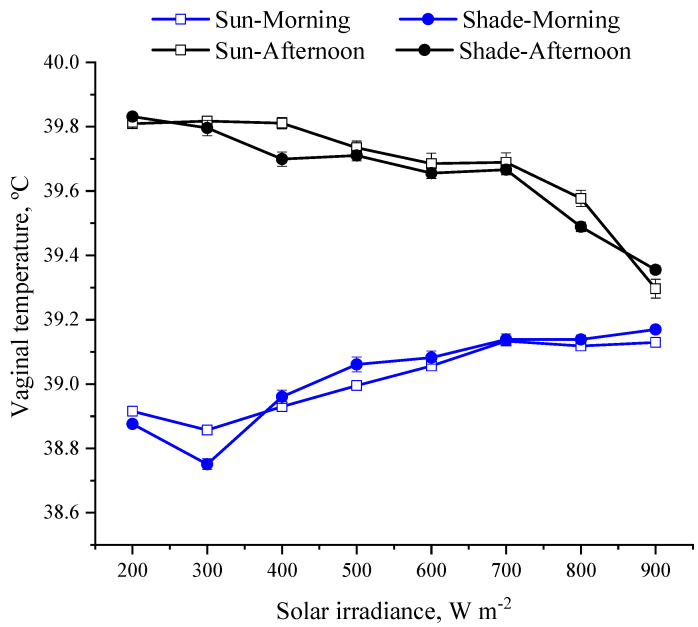
Least square means (±SEM) of vaginal temperatures of heifers when in shade and when exposed to sun.

**Figure 5:**
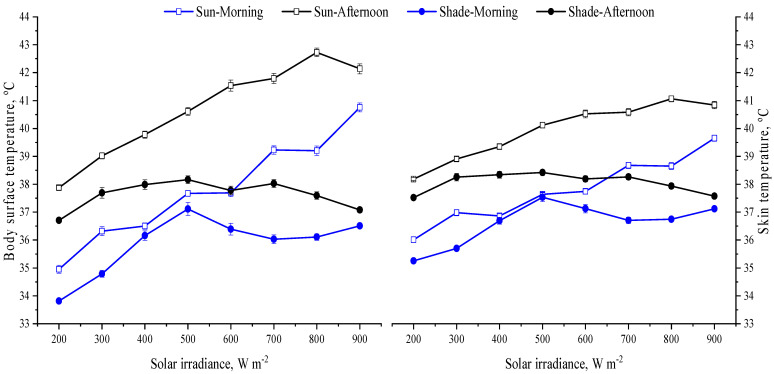
Least square means (±SEM) of body surface and skin temperatures of heifers when in shade and when exposed to sun.

**Figure 6:**
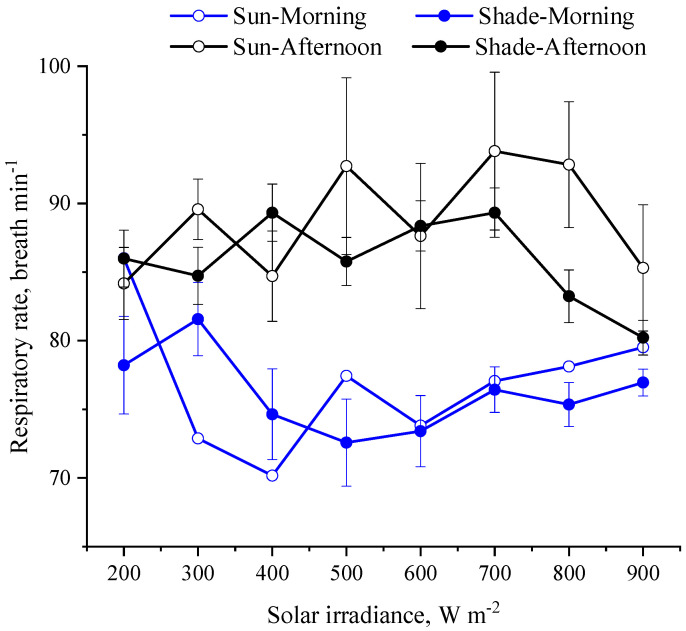
Least square means (±SEM) of respiratory rates of heifers in when they are in shade and when exposed to sun.

This study is the first one to attest that the shade produced by solar panels can efficiently reduce the costs of thermoregulation for animals kept in conditions with high solar inputs (>1000 W m^−2^). Our results revealed that the shade created by the solar panels also helped heifers to keep their core body temperature within a narrow range (~1.2 °C), as indicated by the slight changes in vaginal temperature (Figure 4). As the solar irradiance increased and the heifers were in shade, the body surface temperature dropped by 6 °C, and the skin temperature dropped by 4 °C (Figure 5). This drop in skin temperature is expected to reduce the peripheral vasodilatation and the requirements for recruiting autonomic thermoregulation such as panting [23,24]. As the solar irradiance exceeded 600 W m^−2^, the respiratory rate of the heifers in shade was lower (*p* < 0.05) compared to that when they were exposed to the sun (Figure 6). Similarly, at low solar inputs (<500 W m^−2^), the dairy cows under solar panels maintained lower vaginal temperature compared to the cows under no solar panels [25]. 

**Figure 7:**
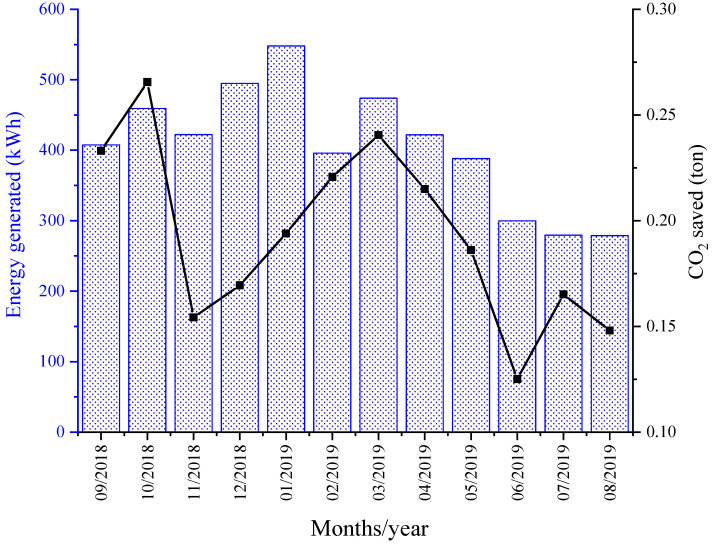
Total monthly electric production by the photovoltaic panels and amount of CO_2_ not emitted to the atmosphere.

**Figure 8:**
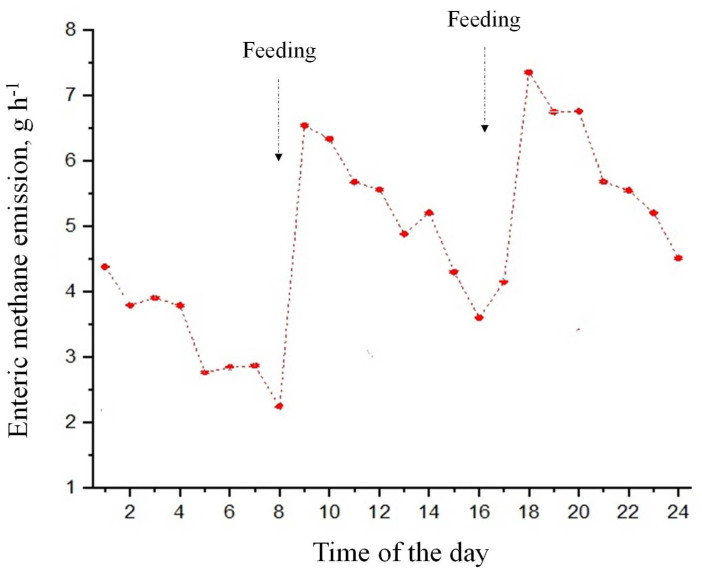
Least square means of enteric methane emission of heifers.

Over a period of one year (from September 2018 to August 2019), a set of ten photovoltaic panels used in this study produced 4869.4 kWh of electricity, which is equivalent to a saving of US $970.00 (Figure 7). The total cost was US $6400.00. The payback time for the photovoltaic panels, which has an expected lifespan of 25 years, is close to seven years. With increasing environmental concerns about greenhouse gas emissions from metabolism and ruminal fermentation, Animal Agrivoltaics has the potential to achieve net-zero emissions, especially for enteric methane (*e*CH_4_) production. The daily *e*CH_4_ emission rate was 63.5 g animal^−1^ or 1.7 kg of CO_2-eq_ (Figure 8). Based on this level of emission, 4.1 m^2^ (nominal power = 335 W) panels per heifer are needed to achieve a net-zero emissions for enteric CH_4_. This area (which ranges between 5 and 9 m^2^) of solar panels represents the optimum shade space requirement for dairy cows [26]. In this study, the solar modules projected 2.7 m^2^ of shade per animal, the offset of which is close to 67% of the total *e*CH_4_ emissions. To achieve a net-zero emissions of *e*CH_4_, therefore, the optimum requirement of shade area needs to be determined.

This study demonstrated that use of shade from solar panels significantly reduced the heat load on heifers, reduced their body temperature and respiration rate, generated renewable electric energy and provided a source of income. The amount of CO_2_ saved by generating electricity by solar panels is sufficient to offset the amount of enteric methane emitted, provided that the optimum amount of shade space is provided. From a comparative standpoint, nutritional strategies through use of feeding additives have the potential to offset only 30% of the enteric methane emissions [27]. Furthermore, if lactating cows have access to shade, the amount of milk production is expected to increase [7,28]. The results suggest that Animal Agrivoltaics in dairy production systems in tropical areas provide improved animal welfare, lower the GHG emissions and provide a source of income.

## 4. Conclusions

1. It can be concluded that Animal Agrivoltaics in dairy systems in tropical climate have the potential and benefit in producing electricity, providing improved thermal comfort for cows, offsetting enteric methane emission to the environment, and become a source of income to farmers.
2. The amount of CO_2_ saved by generating electricity by solar panels is sufficient to offset the amount of enteric methane emitted, provided that optimum amount of shade space is provided.
3. Shade produced by solar panels reduced costs of thermoregulation on animals kept at conditions of high solar input (>1000 W m^−2^). As solar irradiance increased and the heifers were in shade, body surface temperature dropped by 6 °C and skin temperature by 4 °C.

## Figures and Tables

**Table 1:** Meteorological variables measured during the study period.

	24 h		08:00–17:00 h	
Meteorological Variables	Mean	Range	Mean	Range
Air temperature, °C	23.8	12.4–33	28.0	20.4–33.0
Solar irradiance, W m^−2^	219.3	0–1100	553.2	24.5–1100
Relative humidity, %	81.4	35.8–100.00	64.9	35.80–100.00
Wind speed, m s^−1^	0.5	0–3.3	0.9	0–3.3

## Data Availability

The data presented in this study are available on request from the corresponding author.
